# Comparison of the epidermal growth factor receptor protein expression between primary non-small cell lung cancer and paired lymph node metastases: implications for targeted nuclide radiotherapy

**DOI:** 10.1186/1756-9966-29-7

**Published:** 2010-01-22

**Authors:** Chuangzhou Rao, Qiongge Hu, Jianhua Ma, Jian Li, Chen Zhang, Li Shen, Qichun Wei

**Affiliations:** 1 Department of Radiation Oncology, the Second Affiliated Hospital, Zhejiang University School of Medicine, Hangzhou 310009, PR China; 2 Ministry of Education Key Laboratory of Cancer Prevention and Intervention, Zhejiang University School of Medicine, Hangzhou 310009, PR China; 3 Ningbo Second Hospital, Ningbo, Zhejiang, PR China; 4 Ningbo First Hospital, Ningbo, Zhejiang, PR China

## Abstract

**Background:**

The knowledge of Epidermal growth factor receptor (EGFR) expression in metastases of NSCLC was limited. In receptor-mediated targeted nuclide radiotherapy, tumor cells are killed with delivered radiation and therapeutic efficiency is mainly dependent on the receptor expression. Thus, the level and stability of receptor expression in both primary tumors and corresponding metastases is crucial in the assessment of a receptor as target. The goal of this study was to evaluate whether EGFR is suitable as target for clinical therapy.

**Methods:**

Expression of EGFR was investigated immunohistochemically in paired samples of lymph node metastases and corresponding NSCLC primary lesions (n = 51). EGFR expression was scored as 0, 1+, 2+ or 3+.

**Results:**

Positive (1+, 2+ or 3+) EGFR immunostaining was evident in 36 of 47 (76.6%) analysed NSCLC primary tumors, and in 78.7% of the corresponding lymph node metastases. When EGFR expression is classified as positive or negative, discordance between the primary tumors and the corresponding metastases was observed in 5 cases (10.6%). EGFR overexpression (2+ or 3+) was found in 53.2% (25/47) of the NSCLC primary tumors and 59.6% of the corresponding metastases. Nine out of the 47 paired samples (19.2%) were discordant: Only three patients who had EGFR overexpression in the primary tumors showed EGFR downregulation (0 or 1+) in lymph node metastases, while six patients changed the other way around.

**Conclusions:**

The EGFR expression in the primary tumor and the corresponding metastasis is discordant in about 10% of the patients. When overexpression is considered, the discordance is observed in about 20% of the cases. However, concerning EGFR overexpression in the primary tumors, similar expression in the metastases could be predicted with a reasonably high probability, which is encouraging for testing of EGFR targeted nuclide radiotherapy.

## Background

Lung cancer is the leading cause of cancer-related mortality in China and in western countries, approximately thirty percent of all cancer-related deaths are because of lung cancer [1]. Non-small cell lung cancer (NSCLC) accounts for 75-80% of all lung cancers [2]. Of all patients with newly diagnosed NSCLC, 65-75% have advanced, unresectable disease [2,3]. Up to half of patients with NSCLC develop metastases at the time of the initial diagnosis [4], and more patients eventually experience metastases in the course of their disease. For stage III/IV NSCLC, platinum-based combined chemotherapy has been considered as the standard therapeutic modality [5-7]. However, such treatment remains suboptimal with median survival time ranging from 7.4 to 10.3 months [8,9], and the 1-year survival is just around 30%. Although small molecular tyrosine kinase inhibitors (TKIs) against Epidermal growth factor receptor (EGFR), such as gefitinib and erlotinib, have been developed with the hope of improving response to traditional cytotoxic agents, only a limited percentage (12%-27%) of patients seem to benefit from such agents [10-13]. The addition of Cetuximab, an anti-EGFR IgG1 monoclonal antibody, to platinum-based chemotherapy has been regarded as a new standard first-line treatment option for patients with EGFR-expressing advanced NSCLC. However, adding cetuximab to a platinum-based doublet achieved only marginal benefits with an overall survival advantage of 1.2 months (11.3 months vs 10.1 months) compared to chemotherapy alone [14]. Additional therapeutical approaches are clearly needed to improve the survival and the quality of life for patients with recurrent and disseminated NSCLC.

Receptor-mediated tumor targeting nuclide radiotherapy could be another option. In this therapeutic modality, tumor cells are killed with delivered radiation and therapeutic efficiency is mainly dependent on the receptor expression and not whether the receptor function can be blocked or not [15]. Thus, receptor overexpression, together with a similar expression in both the primary tumors and the disseminated lesions, is considered necessary for the success of targeted nuclide radiotherapy.

EGFR is overexpressed in up to 80% of NSCLC [16-18]. However, it is still uncertain whether the EGFR protein expression determined in the primary tumors exactly reflects the EGFR status of the metastatic tumors in NSCLC patients. In the present study, the EGFR expression was investigated immunohistochemically in a series of 51 primary NSCLC samples and corresponding lymph node metastases. The goal was to evaluate whether the receptor is suitable as target for clinical therapy, including radionuclide based therapy.

## Methods

### Patients and Samples

Patients with NSCLC who were treated with curative resection for excision of primary tumor and corresponding lymph nodes metastases, between 2006 and 2007, were enrolled in the present study. Tumor samples from all patients were obtained at the time of operation through the Thoracic Surgery (Oncology) Department and the Pathology Department, Ningbo Second Hospital, under approval of the Institutional Review Board in accordance with the Declaration of Helsinki. Paraffin sections from both the primary tumors and the corresponding lymph node metastases were required for inclusion. Tissue samples were not taken from distant metastases so these were not available for analysis. Patients who had received preoperative thoracic radiotherapy or preoperative systemic chemotherapy were excluded. Patients who had received anti-EGFR therapy were also excluded. Totally, 51 patients were finally included in the study. Clinical information was obtained from the hospital records and included patient age, gender, disease stage, and histological pattern. Lung cancer histology was defined according to the World Health Organization pathology classification [19]. Clinicalpathologic staging was determined according to the International Union Against Cancer tumor-node-metastasis classification of malignant tumors [20]. The patient and tumor characteristics of the analyzed cases are shown in Table 1.

**Table 1:** Tumour and patient characteristics (n = 51)

Characteristics	Patients, n (%)	
Age at diagnosis, years		
Medium	61	
Range	40-78	
		
Gender		
Male	35	(68.6)
Female	16	(31.4)
		
Histology		
Squamous cell carcinomas	18	(35.3)
Adenocarcinomas	27	(52.9)
Bronchioloalveolar carcinoma	2	(3.9)
Adenosquamous carcinoma	4	(7.8)
		
T-stages of the primary lesions		
T1	8	(15.7)
T2	32	(62.7)
T3	5	(9.8)
T4	6	(11.8)
		
N-stages		
N1	20	(39.2)
N2	28	(54.9)
N3	3	(5.9)
		
M-stages		
M0	46	(90.2)
M1	5	(9.8)
		
Stages at diagnosis		
II	13	(25.5)
IIIA	29	(56.9)
IIIB	4	(7.8)
IV	5	(9.8)

### EGFR-staining

The tissues were fixed in 4% buffered formalin, processed and embedded in paraffin. Sections, 4-μm thick, were then cut and deparaffinized in xylene and hydrated through graded concentrations of ethanol to distilled water. EGFR was assessed by immunohistochemistry as previously described [21]. Briefly, after deparaffinization of the sections, endogenous peroxidase was blocked in 0.3% H_2_O_2 _in PBS for 20 min. For antigen retrieval, the sections were submitted to high temperature and pressure with Tris-EDTA buffer (pH 9) for 5 min. The slides were preincubated in PBS for 10 min. The primary mouse monoclonal antibody directed against EGF receptor (clone 31G7, Zymed labs, South San Francisco, CA, USA) receptor was diluted 1:100, and incubated overnight at 4°C. The secondary biotinylated antibodies (goat anti-mouse from Dako, Glostrup, Denmark) and the peroxidase-labelled streptavidin-biotin complex (Dako) were diluted 1:200 and incubated for 30 min at room temperature. All slides were developed in 0.05% diamino benzidine (Sigma, St Louis, MO, USA) for 5 min and counterstained in Harris haematoxylin (Sigma). Finally, the slides were dehydrated through graded alcohol to xylene and mounted in organic mounting medium.

### EGFR-scores

EGFR stainings were mainly in the cell membranes and the expression pattern of EGFR was quite similar to that of HER2. Thus EGFR expression was therefore evaluated using the HercepTest scoring criterion as reported in previous studies [21-23]. Sections were considered as positive when at least 10% of the tumor cells to be stained. Cytoplasmic staining without associated membrane staining was considered non-specific and was reported as negative. The score was based on a scale where 0 corresponded to tumor cells that were completely negative, 1+ corresponded to faint perceptible staining of the tumor cell membranes, 2+ corresponded to moderate staining of the entire tumor cell membranes and 3+ was strong circumferential staining of the entire tumor cell membranes creating a fishnet pattern. As positive controls we used in house positive control tissue sections. As negative controls we used normal tissues, which are expected not to express EGFR such as connective tissue seen in the same sections as the tumor cells. In the metastases sections we used lymphocytes and the surrounding capsule of the lymph nodes as negative internal controls.

### Excluded cases

In 3 cases, no tumor cells could be found in the sections of lymph nodes. In another case, there were no tumor cells in the sections supposed to be primary lung cancer. Thus, we started from 51 patient cases and ended up with 47 cases with high quality material of both primary tumors and the corresponding metastases.

## Results

### EGFR expression of primary tumors and metastases

The EGFR-scores for the analyzed 47 primary NSCLC and the corresponding 47 lymph node metastases are shown in Table 2. In 36 of 47 (76.6%) analysed primary tumors, immunostaining for EGFR was evident. Among these, 11 (23.4%) had EGFR expression scored as 1+, 10 (21.3%) had EGFR expression scored as 2+, and 15 (31.9%) had EGFR expression scored as 3+. Accordingly, negative EGFR staining was seen in 11 cases (23.4%) of the analysed primary tumors. Positive EGFR expression (1+, 2+ or 3+) was found in 78.7% (37/47) of the corresponding lymph node metastases, the cases with EGFR expression scored as 0, 1+, 2+ or 3+ were 10 (21.3%), 9 (19.1%), 18 (38.3%), and 10 (21.3%) respectively.

**Table 2:** EGFR-scores for the analyzed primary Non-small cell Lung cancer and the corresponding lymph node metastases (n = 47).

Primary tumor EGFR-scores	Lymph node metastases EGFR-scores			
	0	1+	2+	3+
0	8	2	1	0
1+	1	5	4	1
2+	0	1	9	0
3+	1	1	4	9

The scoring was based on a scale where 0 corresponded to completely negative staining, 1+ corresponded to faint perceptible staining of the tumor cell membranes, 2+ corresponded to moderate staining of the entire tumor cell membranes and 3+ was strong circumferential staining of the entire tumor cell membranes creating a fishnet pattern

**Figure 1:**
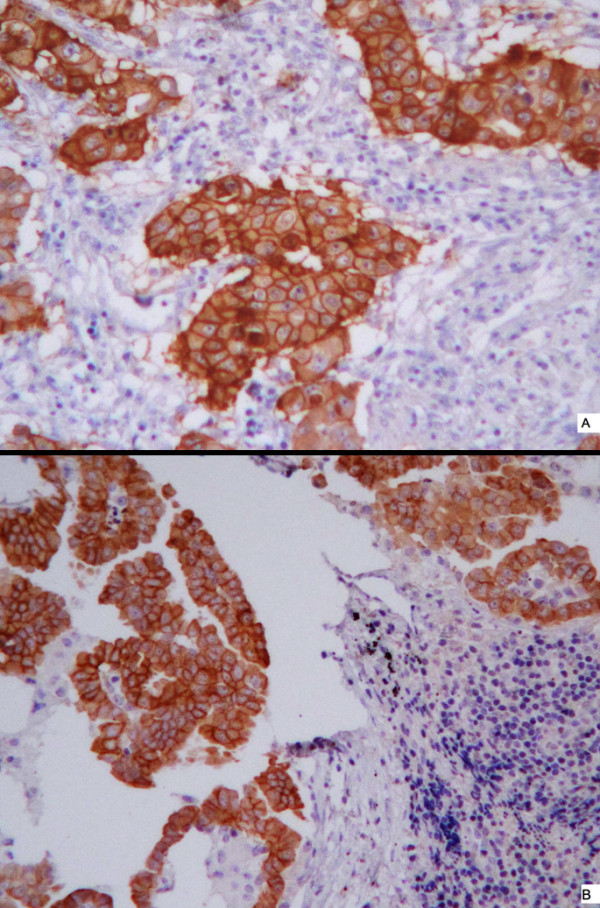
**Comparisons of immunohistochemical EGFR staining of primary non-small cell lung cancer (A) and corresponding metastases (B)**. Both A and B (from the same patient) were scored 3+. The micrographs were taken with objective × 40.

EGFR overexpression (2+ or 3+) was found in 53.2% (25/47) of the NSCLC primary tumors and 59.6% (28/47) of the corresponding lymph node metastases. Example of staining pattern for a primary tumor and the corresponding metastasis (which both were scored as 3+) is shown in Fig. 1A and 1B.

### Comparison of the EGFR status between primary tumors and metastases

When EGFR expression is classified as positive (1+, 2+ or 3+) or negative, a discordance was observed in 5 cases (10.6%): in 2 cases, EGFR was expressed in the primary tumor but not in the metastasis, while three samples showed EGFR expression in the metastasis but not in the primary tumor. There was a good agreement between the primary tumors and the corresponding lymph node metastases in the majority of cases. EGFR expression retains or gains in the metastases in more than 95.7% (45/47) of the cases.

Regarding EGFR overexpression, nine out of the 47 paired samples (19.2%) were discordant for EGFR status between the primary site and the metastases: only three patients who had 2+ or 3+ in the primary tumors and changed to 0 or 1+ in lymph node metastases, and another six patients who had 0 or 1+ in the primary tumors and changed to 2+ or 3+ in lymph node metastases. The major results from the EGFR-score analyses are summarized in Table 3.

**Table 3:** Major results from the EGFR-scores analyses of non-small cell lung cancer (n = 47).

EGFR-scores characteristics	Cases	%
Primary tumors with 2+ or 3+	25	(53.2)
Lymph node metastases with 2+ or 3+	28	(59.6)
Unchanged EGFR-scores in lymph node metastases vs. the primary tumor	31	(66.0)
Changed EGFR-scores in lymph node metastases vs. the primary tumor	16	(34.0)
Patients who had 0 or 1+ in primary tumors and changed to 2+ or 3+ in lymph node metastases	6	(12.8)
Patients who had 2+ or 3+ in primary tumors and changed to 0 or 1+ in lymph node metastases	3	(6.4)
Patients who had 0 in primary tumors and changed to 1+, 2+ or 3+ in lymph node metastases	3	(6.4)
Patients who had 1+, 2+ or 3+ in primary tumors and changed to 0 in lymph node metastases	2	(4.2)

## Discussion

The knowledge of EGFR expression in metastases of NSCLC was limited. It is still unclear whether the metastases lose, gain or retain the receptor status relative to the primary tumors. For a receptor to be of interest for targeting, a similar expression in both the primary tumors and the disseminated lesions are required. Investigation into the receptor status between metastases and the primary tumors will provide valuable information on whether the receptor is suitable as a target for diagnostic and/or therapeutic procedures. In the present study, the expression of EGFR was investigated immunohistochemically in paired samples from a series of primary NSCLC lesions and corresponding lymph node metastases.

EGFR expression (1+/2+/3+) was found in 76.6% of the primary lesions and 78.7% of the lymph node metastases. EGFR expression in NSCLC cancer has been reported to be common (ranges from 40-80%) [16-18]. Our result is consistent with the former findings of high EGFR expression in NSCLC [24,25]. Moreover, the frequency of EGFR expression in lymph node metastases was approximately as high as in the primary lesions of NSCLC.

It is known that EGFR is commonly expressed in normal cells. When EGFR targeted radionuclide therapy is delivered, possible side effects to normal tissues should be taken into consideration. It might be possible to minimize the toxicity and improve therapeutic efficiency if a tumor and its metastases have a strong EGFR expression to ensure higher tumor uptake than in most normal tissues. So, EGFR overexpression (2+ or 3+) was also analysed in the present study. EGFR overexpression was found in 53.2% of the NSCLC primary tumors and 59.6% of the corresponding lymph node metastases.

To our knowledge, the question of EGFR protein expression in metastases versus primary NSCLC, has not been well addressed. Although totally 16 changes were observed in the present study, switch from positive EGFR expression in the primary tumor to negative in the metastatic site was observed only in 2 cases (4.2%, 2/47) and negative to positive EGFR conversions occur less than 6.5% of the cases (3/47). When overexpression is considered, a discordance was observed in 19.2% of the cases: only 3 patients with EGFR overexpression in the primary tumor had lower EGFR scores in the corresponding lymph node metastases. Moreover, in another 6 patients, EGFR overexpression was gained in lymph node metastases while the primary tumors had low scores. Although the current report is limited by the small sample size, our observations suggest that positive EGFR expression is relatively well-preserved during the metastatic progression from primary NSCLC to lymph node metastases. Therefore, concerning positivity, the EGFR expression determined in the primary tumor is predictive for the metastases. Recent studies on laryngeal, esophageal, and uterine cervical carcinoma also found that the EGFR status of the primary tumor was retained in the metastases [21-23].

There are few reports in the literature concerning the stability of EGFR protein expression between paired samples of NSCLC primary tumors and the corresponding metastases. In the studies by Italiano et al [26] and Gomez-Roca et al [27], analyzed by immunohistochemistry, 33% of the cases with NSCLC showed discordance in EGFR status between primary tumor and metastases, suggesting that EGFR expression might not be stable during metastasis progression. However, according to the recent report by Badalian et al, the expression status of EGFR protein was reported to be highly similar in the bone metastasis compared to that in primary NSCLC, without positive to negative or negative to positive EGFR conversions occur in their small cohort of NSCLC [28]. Individual comparison of corresponding primary and metastatic tissues indicated that downregulation of EGFR was a rare event (2/11 cases) while upregulation was observed more frequently (4/11 cases), however, the expression level was maintained in about half of the analyzed cases. This observation suggests that EGFR expression status is relatively well-preserved during metastatic progression of NSCLC to the bone. In another study, Milas et al [18] reported on analysis of EGFR expression in 29 cases NSCLC with brain metastases. Nine out of the 29 cases were studied regarding EGFR expression in the lymph node metastases. Immunostaining was present in 84% (21/25) of the primary tumors, in 56% (5/9) of the lymph nodes metastases, and in 59% (17/29) of the brain metastases. However, comparisons of paired samples from primary tumors and corresponding metastases were not made. There are conflicting results regarding the stability of EGFR protein expression between paired samples of NSCLC primary tumors and the corresponding metastases, and our research add to the body of data on the subject.

## Conclusions

The EGFR is commonly expressed in NSCLC, its expression in the primary tumor and the corresponding lymph node metastasis is discordant in about 10% of the patients. When overexpression is considered, the discordance is observed in about 20% of the cases. However, concerning EGFR overexpression in the primary tumors, similar expression in the metastases could be predicted with a reasonably high probability, which is encouraging for testing of EGFR targeted nuclide radiotherapy.

## Competing interests

The authors declare that they have no competing interests.

## Authors' contributions

CR and QH participated in the design of the study, carried out the clinical and immunohistochemical data analysis; JM and LS interpreted the histological and immunohistochemical data; JL and CZ contribute with the clinical data; and QW conceived the study, interpreted the immunohistochemical data and wrote the manuscript. All authors read and approved the final manuscript.
